# Synergistic effects of organic fertilizer and copper-calcium nanoparticles on onion growth and storage under arid conditions

**DOI:** 10.1038/s41598-026-57028-w

**Published:** 2026-06-23

**Authors:** Abeer Abd EL Moiez Ahmed Bakr, Sobhi F. Lamlom, Abdel-Haleem A. H. El-Shaieny, Ahmed M. Abdelghany, Reham M. Abdalla

**Affiliations:** 1 Soil and Water Department, Faculty of Agriculture, Qena University, Qena, Egypt; 2 Plant Production Department, Faculty of Agriculture Saba Basha, Alexandria University, 21531 Alexandria, Egypt; 3 Department of Horticulture, Faculty of Agriculture, Qena University, 83523 Qena, Egypt; 4 Crop Science Department, Faculty of Agriculture, Damanhour University, Damanhour, Egypt; 5 Vegetable Crops Department, Faculty of Agriculture, Assiut University, Assiut, 71526 Egypt

**Keywords:** *Allium cepa*, Organic amendment, Arid agriculture, Post-harvest quality, Sustainable agriculture, Environmental sciences, Plant sciences

## Abstract

Onion (*Allium cepa* L.) production in hyper-arid environments is constrained by poor soil fertility, limited water availability, and post-harvest losses that reduce market viability. The interactive effects of foliar application of copper oxide (CuO) and calcium oxide (CaO) nanoparticles (NPs), combined with sugarcane filter mud cake (FMC), on onion growth and storage quality in calcareous hyper-arid soils remain to be characterized. A two-season field study (2023/24–2024/25) evaluated the effects of FMC (30 t ha⁻¹) combined with foliar CuO NPs (10, 20, and 30 mg L⁻¹) and CaO NPs (50, 100, and 150 mg L⁻¹), applied singly and in combination, on onion cv. Sabeeni in Upper Egypt. Vegetative growth, yield traits, nutrient uptake, and storage performance were quantified. Organic fertilization and nanoparticle treatments interacted significantly: yields of 42–45 t ha⁻¹ (15–21% above control) were achieved with low-to-moderate CuO NPs (10–20 mg L⁻¹) and high CaO NPs (150 mg L⁻¹); high CaO NPs alone maintained yields close to control values (< 3% increase); and excessive CuO NPs (30 mg L⁻¹) with insufficient Ca was associated with phytotoxicity and yield reductions (21.29–28.90 t ha⁻¹). Tissue Cu concentrations ranged from 6.17 to 39.33 mg kg⁻¹ and Ca from 2,556 to 4,750 mg kg⁻¹. These results suggest that combining organic amendment with appropriately balanced nanoparticle nutrition may represent a scalable approach to improving onion yield by 15–21% and extending post-harvest shelf life under hyper-arid conditions.

## Introduction

Ranked second among vegetable crops globally by production volume, onions (*Allium cepa* L.) are grown on about 5.2 million hectares worldwide with an annual production of over 105 million tons^1^. Beyond their nutritional value, onions represent an economically significant crop in both developed and developing countries^2–5^, with Egypt among the principal producers and exporters to European and Middle Eastern markets. Declining soil fertility, low nutrient use efficiency (NUE), postharvest losses exceeding 30–40%, and volatile market prices continue to constrain farm income^6,7^.

Onion has specific nutritional requirements that vary across growth stages, with macronutrient demand increasing during bulb development. While primary nutrients have received more research attention, the role of secondary nutrients and micronutrients, particularly Ca and Cu, is receiving greater attention^8^. Ca contributes to cell wall and membrane stability and functions as a second messenger in signal transduction pathways that mediate responses to environmental stress^9^. Cu is an essential cofactor for various functions, including photosynthetic electron transport (plastocyanin), respiration (cytochrome c oxidase), and antioxidant defenses^10,11^. Conversely, conventional fertilization methods are not efficient in correcting micronutrient deficiencies because calcareous arid soils have a higher pH (> 7.5), which substantially decreases nutrient solubility and bioavailability^12^.

Nanotechnology has been proposed as an approach to improving nutrient supply efficiency in agriculture, with potential implications for fertilizer use and environmental management^13^. Nanoparticles (1–100 nm) possess unique physicochemical properties compared to their bulk counterparts, including a high surface area-to-volume ratio, enhanced cellular uptake, and controlled release processes^14^. Application of nanoparticles on plant leaves circumvents shortcomings related to fixation, leaching, and microbial immobilization, which reduce the functionality of nutrients applied via conventional soil^15^. CuO and CaO NPs are known to enhance photosynthesis, antioxidant enzyme activity, and bolster cell walls in a variety of horticultural crops^16^.

Combining organic amendments with nanoparticle-based nutrition may address multiple production constraints simultaneously^17^. Organic agri inputs enhance soil physical properties, increase cation exchange capacity, improve water-holding capacity, and release nutrients over time in doses low enough to support slow growth^18,19^. Filter mud cake (FMC), a nutrient-rich byproduct of sugarcane juice clarification with near-neutral pH, is widely available in areas adjacent to sugar mills^20–22^, and has recently been assessed as an organic amendment. Its high organic matter content and phosphate retention capacity may improve nutrient use efficiency and potentially reduce the inputs required from foliar nanoparticle applications to sustain crop yield and quality^23^. Post-harvest quality and storage stability are economically important dimensions of onion production that receive insufficient research attention in many production regions. Sprouting, pathogen-related decay, and physiological deterioration during storage contribute to market price instability and income insecurity among smallholder farmers in subtropical regions, underscoring the need for post-harvest research^24^. In particular, Ca plays a key role in maintaining bulb firmness and can hamper senescence and decrease susceptibility to diseases by strengthening the middle lamella^25^. Cu has antimicrobial activity and participates in lignin synthesis, which may make storage performance better^26^.

The interactive effects of organic fertilization and combined CuO-CaO NPs treatments on onion production under hyper-arid conditions have not been characterized. Prior studies have examined organic amendments or nanoparticle nutrition in isolation, leaving open questions about whether organic matter modification affects nanoparticle uptake efficiency, Cu-Ca antagonism, or post-harvest quality. This study tested three hypotheses: (1) that FMC organic amendment enhances nanoparticle efficacy by improving plant health and soil physicochemical properties; (2) that balanced Cu-Ca nutrition produces effects greater than those observed for either element alone; and (3) that pre-harvest nutritional management improves bulb mineral content and post-harvest storability. The study objectives were to evaluate growth and physiological responses, identify effective nanoparticle formulations for onion yield and quality traits, and characterize metal accumulation profiles in vegetative and storage tissues. Two growing seasons were used to reduce the influence of year-to-year environmental variation.

## Materials and methods

### Experimental site and climate

Field experiments were carried out across two winter growing seasons (2023/24 and 2024/25) on the grounds of the Agricultural Experimental Farm, Faculty of Agriculture, Qena University, Egypt (26°11′ 25″ N, 32°44′ 42″ E). This extremely hot and arid desert bordering the Tropic of Cancer receives less than 5 mm annual winter rainfall, receives an average solar radiation ranging from 18 to 22 MJ m⁻² day⁻¹^27^. Night temperatures from November to March typically ranged from 12 °C to 28 °C, while relative humidity ranged from 33% to 58%. These climate conditions are characteristic of an arid subtropical climate found in Upper Egypt, where onion is an important winter crop.

### Soil characterization

The soil used in the experiment was a sandy loam based on the USDA taxonomy. Composite samples were collected from 0 to 30 cm depth in the field at 15 random points before planting, air-dried, ground, and sieved through a 2 mm sieve. The samples were dispersed with sodium hexametaphosphate (NaPO_2_), and particle-size distribution was measured by the pipette method, yielding 62.7% sand, 22.1% silt, and 15.2% clay (Table 1). Soil pH was determined in a 1:1 soil-water suspension using a glass-electrode pH meter, yielding 8.19, moderately alkaline, characteristic of calcareous arid soils. EC was measured at 3.22 dS/m in the 1:10 soil-water extract, indicating slight salinity. Organic matter was 0.69% (Walkley-Black wet oxidation method), typical of sandy, arid soils with limited organic inputs. Pigment content was calculated by volumetric method according to the Collins calcimeter, ca. carbonate 8,59% in the skin; Table 1. Nutrients available (total nitrogen at 0.12 g/kg by micro-Kjeldahl digested; available phosphorus by Olsen’s sodium bicarbonate extraction measured at 6.55 mg/kg; and exchangeable potassium extracted with 1 N ammonium acetate (pH = 7·0) and analyzed using flame photometry, with values expressed as mg/kg). The available Cu and Ca contents were determined using the DTPA-leaching procedures according to Williams & Steinberg (1959): 10 g of soil samples was shaken with 20 mL of a DTPA solution (0.005 M DTPA, 0.01 M CaCl₂, 0.1 M triethanolamine, pH = 7.3) for two hours at a temperature of 25 °C using a shaking machine followed up by filtration through filter paper [15]. Cu and Ca concentrations were measured from atomic absorption spectrophotometry readings at 324.8 nm and 422.7 nm, respectively. A 1% wt/v solution of LaCl₃ was added to standards and samples to minimize phosphate interference. Quality control included the use of certified reference materials (NIST SRM 2709), method blanks, and duplicates with recovery rates of all elements between 95 and 105%.

**Table 1:** Soil analysis before cultivation.

Property	Value
Particle size distribution:	
Sand (%)	62.70
Silt %)	22.10
Clay (%)	15.20
Texture class	Sandy loam
pH (1:1) in water	8.19
ECe (dS m⁻¹)	3.22
Ca carbonates (%)	8.59
Organic matter (%)	0.69
Total (N) g/Kg	0.12
Total P g/Kg	0.25
Total K g/Kg	0.21
Available P (mg/Kg)	6.55
Available K (mg/Kg)	76.88

### Nanoparticle characterization and preparation

**Fig. 1:**
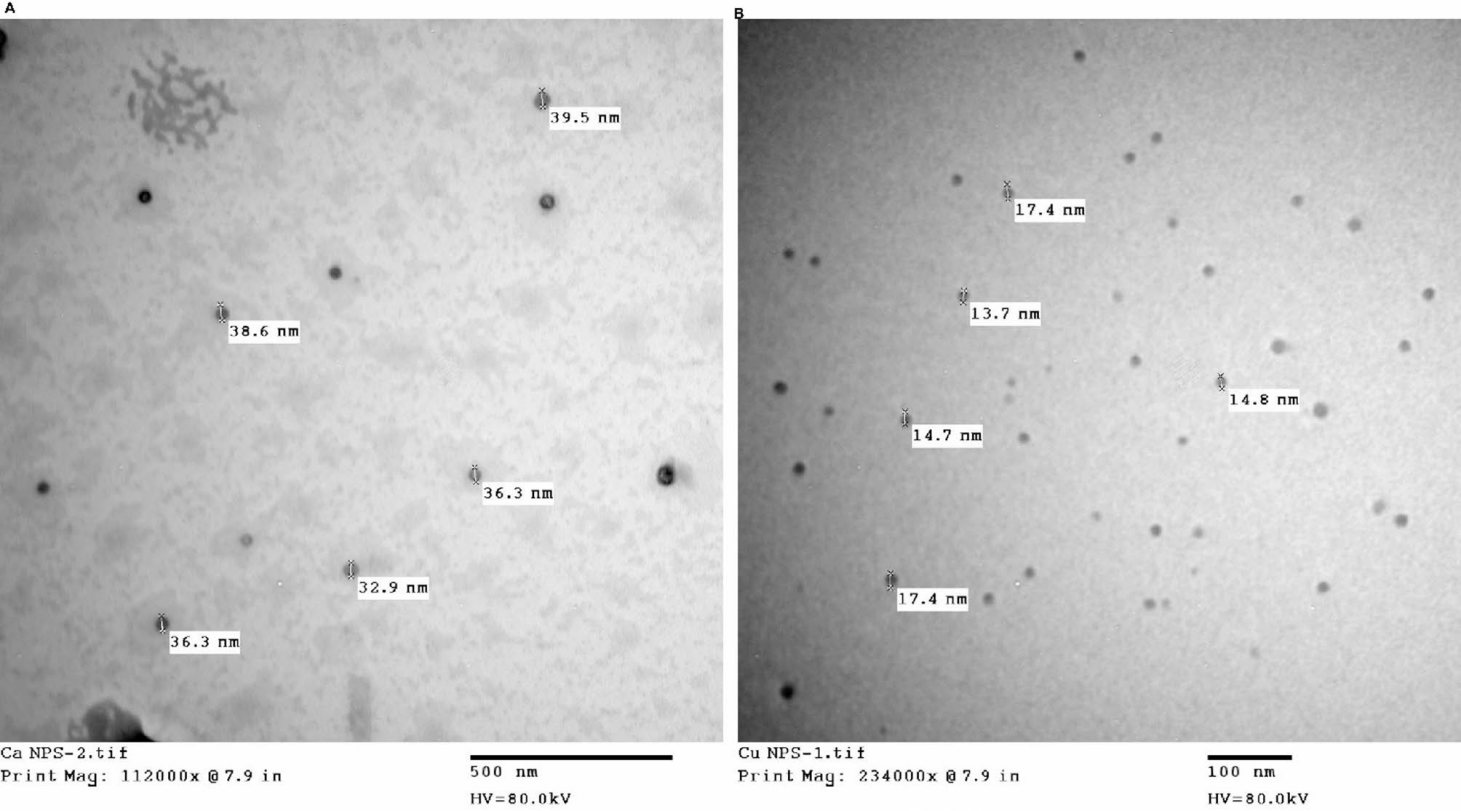
Transmission electron microscopy (TEM) characterization of synthesized nanoparticles. (A) High-resolution TEM micrograph of CaO NPs at 112,000× magnification showing spherical particles with uniform morphology. (B) High-resolution TEM micrograph of CuO NPs at 234,000× magnification, showing smaller, well-dispersed spherical particles with a tight size distribution. Scale bar = 100 nm.

Nanoparticle morphology, size distribution, and crystallinity were analyzed using transmission electron microscopy (TEM) with a Tecnai G2 F20 at 80 kV. The experimental process involved suspending the nanoparticles in absolute ethanol, dropping them onto carbon−coated copper grids, and air-drying at room temperature to evaporate the solvent. Morphology of CaO NPs, and CuO NPs are captured using high-resolution micrographs at 234,000× magnifications for the former sample (CuO NPs),168000x scale bar, distinct representative samples to allow quantification via ImageJ software, with a minimum of thirty particle diameter measurements per sample necessary to obtain average size, including all diameters divided by units into a frequency histogram plot, and distributions respectively in cumulative bin presentations. CuO NPs presented as a homogenous spherical shape with diameters from 13.7 to 17.4 nm (average ± SD:15.2 ± 1.6 nm, *n* ≥ 30) (Fig. 1A). Initial conditions resulted in CaO NPs that were bimodally distributed with a spherical particle at 51.2 nm and an elongated particle at a mean size of (61.9 × 26.4) nm, while modified shape conditions synthesized more uniform spheres from ca. oxides within the range sizes between 32.9 − 39-(mean ± SD:36 − 3±−28-nm n = C≥30) (Fig. 1B). For plant experiments, nanoparticles at 50, 100, and 200 mg L⁻¹ were dispersed in distilled water (D. w.) and sonicated in an ultrasonic bath at 40 kHz for 15 min to reduce agglomeration and clump formation upon re-suspension. Stability and agglomeration during storage and use were monitored using dynamic light scattering (DLS) in a Zetasizer Nano ZS. The nanoparticle preparations were autoclaved at 121 °C and 15 psi for 20 min to sterilize the solutions and prevent microbial contamination, thereby ensuring that plant health is not compromised during subsequent experiments.

### Experimental design

A two-factor randomized complete block design with three replications was employed, totaling 96 plots. Each plot measured 10.5 m² (3.5 m × 3.0 m) and contained five rows spaced 50 cm apart with 10 cm within-row spacing, supporting 150 plants per plot. Factor A comprised two organic fertilizer management systems (with and without FMC at 30 t ha⁻¹ dry weight). Factor B encompassed sixteen nanoparticle treatments: control (mineral fertilizer only), three CuO NPs concentrations (Cu1: 10 mg L⁻¹, Cu2: 20 mg L⁻¹, Cu3: 30 mg L⁻¹), three CaO NPs concentrations (Ca1: 50 mg L⁻¹, Ca2: 100 mg L⁻¹, Ca3: 150 mg L⁻¹), and nine CuO-CaO NPs combinations (Cu1Ca1, Cu1Ca2, Cu1Ca3, Cu2Ca1, Cu2Ca2, Cu2Ca3, Cu3Ca1, Cu3Ca2, Cu3Ca3)^28^(Table 2). Each nanoparticle treatment was tested under both organic fertilizer management systems, creating 32 unique treatment combinations across two seasons.

**Table 2:** Treatment combinations of CuO and CaO NPs with organic fertilizer management systems.

Factor A: Organic Fertilizer Management			
Without organic			
With organic (FMC) ^**†**^	Filter mud cake at 30 t ha⁻¹ dry weight		
**Factor B: Nanoparticle Treatments**			
**Treatment Code**	**CuO (ppm)**	**CaO (ppm)**	**Description**
Control	0	0	Mineral fertilizer
Cu1	10	0	Low Cu alone
Cu2	20	0	Medium Cu alone
Cu3	30	0	High Cu alone
Ca1	0	50	Low Ca alone
Ca2	0	100	Medium Ca alone
Ca3	0	150	High Ca alone
Cu1Ca1	10	50	Low Cu + low Ca
Cu1Ca2	10	100	Low Cu + medium Ca
Cu1Ca3	10	150	Low Cu + high Ca
Cu2Ca1	20	50	Medium Cu + low Ca
Cu2Ca2	20	100	Medium Cu + medium Ca
Cu2Ca3	20	150	Medium Cu + high Ca
Cu3Ca1	30	50	High Cu + low Ca
Cu3Ca2	30	100	High Cu + medium Ca
=	30	150	High Cu + high Ca

†Filter mud cake (FMC) composition: 58.3% organic matter, 1.85% N, 0.92% P, 1.34% K, 2.15% Ca, 28.5 mg kg⁻¹ Cu, C:N ratio 18.3:1, pH 7.2, EC 3.8 dS m⁻¹. ±Each nanoparticle treatment (Factor B) was applied under both organic fertilizer management systems (Factor A), resulting in 32 distinct treatment combinations tested across the two growing seasons (2023/24 and 2024/25).

### Plant material and cultural practices

The onion cultivar Sabeeni was selected for its agronomic and logistical suitability to the study region. It is the dominant commercial variety in Upper Egypt, accounting for approximately 60% of the regional onion cultivation area, and is better adapted to hot, dry conditions than other commonly grown Egyptian cultivars such as Giza 6 and Giza 20. When properly cured, Sabeeni has a storage life of 4–6 months under ambient conditions, making it appropriate for evaluating post-harvest quality in response to nutritional treatments. The cultivar is also moderately responsive to fertilizer application in regional trials, supporting its use as a test genotype. Use of a single cultivar minimized genotypic variation and improved sensitivity for detecting treatment effects, though caution is warranted in extrapolating specific quantitative responses to other germplasm. Seeds were sown in prepared nursery beds in mid-September using material obtained from the Horticultural Research Institute (Agricultural Research Center, Giza, Egypt). Seedlings with 6–8 leaves and a stem diameter of 5–6 mm were transplanted in early November (45 days after sowing) each season. Standard agronomic practices followed the Ministry of Agriculture guidelines for Egypt. Furrow irrigation was applied every 7–10 days throughout the vegetative stage and was withheld 15 days before harvest. Weed management consisted of a pre-emergence application of pendimethalin (1.0 kg a.i. ha⁻¹, 2 days after transplanting), supplemented by two hand weedings at 35 and 65 DAT. Onion thrips (Thrips tabaci) were monitored weekly using yellow sticky traps; spinosad (144 g a.i. ha⁻¹) was applied when populations exceeded the economic threshold of 5 thrips per plant. Purple blotch (Alternaria porri) and downy mildew (Peronospora destructor) were managed preventively with azoxystrobin (250 g a.i. ha⁻¹), chosen specifically to avoid confounding from Cu-containing fungicides. Pest and disease management measures were applied uniformly across all treatments, and application dates were recorded for each plot.

### Organic fertilizer application

FMC contained 653.4 g kg⁻¹ organic matter, 23.1 g kg⁻¹ N, 25.1 g kg⁻¹ P, 3.2 g kg⁻¹ K, with a C: N ratio of 14.5:1, pH 6.7, and EC 5.6 dS m⁻¹ (Table 3). FMC was incorporated at a rate of 30 t ha⁻¹ dry weight two weeks prior to transplanting, mixed into the soil to a depth of 20–25 cm to ensure even distribution and early nutrient release.

**Table 3:** Filter Mud Cake (FMC) properties.

Properties	Filter Mud Cake (FMC)
pH (1:1)	6.70
EC (1:10) (dS m⁻¹)	5.60
OM (g kg^− 1^)	653.40
Organic carbon (g kg^− 1^)	379.00
Total nitrogen (g kg^− 1^)	23.10
C/N ratio	14.50
Total P (g kg^− 1^)	25.10
Total K (g kg^− 1^)	3.20

### Nanoparticle application

Foliar nanoparticle sprays were applied at 30, 45, 60, and 70 days after transplanting (DAT), corresponding to vegetative establishment (i), early bulbing (ii), rapid bulb growth (iii), and late bulb maturation (iv). Fresh suspensions were prepared immediately before each application by dispersing CuO and CaO NPs in deionized water containing 0.05% (v/v) Tween-20 surfactant. Suspensions were sonicated for 15 min at 40 kHz to prevent agglomeration. Applications were made in calm conditions (wind speed < 5 km/h; temperature 18–22 °C; relative humidity 65–75%) during the early morning (06:00–08:00). A manually operated 15 L knapsack sprayer (Solo model 425) with an adjustable hollow-cone nozzle was calibrated to deliver 400 L ha⁻¹ at 2.5–3.0 bar, applied to both leaf surfaces to the point of runoff. A temporary polyethylene barrier (1.5 m height) was installed between plots during each spray event to minimize cross-contamination. Total Cu applied per season across four spray events ranged from 40 g ha⁻¹ (Cu1) to 80 g ha⁻¹ (Cu2) and 120 g ha⁻¹ (Cu3). These application rates are substantially lower than those used with conventional Cu fungicides (2–5 kg Cu ha⁻¹ per season) and well below the EU limit for Cu in organic farming (6 kg Cu ha⁻¹ annually).

### Measured parameters

#### Vegetative growth parameters

At 70 days after transplanting, three plants were randomly chosen from the central rows of each plot (excluding border plants), and samples were obtained for morphological measurements. Considered intact, they were washed by first shaking in a tap-water solution, then rinsing and shaking the clips for 30 s each in two additional batches of deionized water before measuring. Fresh weight was measured using a precision balance (± 0.01 g). To determine dry weight, samples were dried at 70 °C for 48–72 h and weighed once the mass remained stable. Plant height (from the soil surface to the tip of the longest leaf), bulb diameter at 70 days (at the widest point), and leaf chlorophyll content (using a SPAD-502Plus meter, mean value from three readings per plant). From each plot, three plants were randomly selected to form technical subsamples (i.e., individual replicates within plots, with their values averaged for analysis), allowing variability within the plot to be measured separately.

#### Yield components

When physiological maturity was reached (about 50% foliage lodging), harvesting was performed at about 150–160 days after transplanting. The total yield of all plants from each plot (except border rows) was measured by uprooting, cleaning, topping (to a neck height of approximately 2–3 cm), and weighing. Marketable yield included only bulbs without defects, such as splits, doubles, disease symptoms, or those smaller than 5 cm in diameter. Total and marketable yields were measured per plot and converted to tons/h hectare.

#### Nutrient content

Samples of dried plants were ground using stainless-steel mills (1-mm mesh) and then stored in sealed containers at room temperature in the dark until analysis. Duplicate tests were conducted for each test. Total nitrogen (after 70 days in dried tissue) was determined by the micro-Kjeldahl method according to AOAC protocols: 0.5 g subsamples were digested with sulfuric acid and a catalyst mix (K₂SO₄, CuSO₄, Se), cooled, diluted, and distilled; ammonia was absorbed in boric acid solution, then titrated with hydrochloric acid. We derived total nitrogen uptake per plant by multiplying the nitrogen concentration by plant dry weight, then scaling it to the field level using experimental spacing. In mineral analysis, 0.5 g of ground tissue was suspended in sulfuric acid and hydrogen peroxide, then heated on a hot plate, cooled, washed twice, resuspended in volumetric flasks, and diluted with deionized water. Copper and calcium contents were determined by atomic absorption spectrophotometer (PerkinElmer Analyst 400) at 324.8 nm and 422.7, respectively, all diluted with standards prepared from the stock solution of 1,000 mg L⁻¹ and expressed as mg kg⁻¹ dry weight. To suppress phosphate interference in calcium analysis, lanthanum chloride (1% w/v) was added. Lachsite quality controls were performed with certified reference materials, blanks and duplicates, resulting in 95–105% recovery rates.

#### Storage quality parameters

For storage experiments, bulbs were harvested, and uniform, disease-free bulbs with a diameter of 6–8 cm were selected. The bulbs were dried in the shade with plenty of airflow for 10–14 days, until the outer scales were dry and papery. Approximately 5 kg of bulbs per treatment was stored in wooden boxes with ventilation holes at ambient temperature (20–25 °C, 60–70% relative humidity), out of the sun and rain. Quality indicators were evaluated monthly: sprouting (> 5 mm shoot emergence), rotting (evidence of decaying tissue), weight loss (> 20% of initial weight), and shriveling and firmness. Data on storage quality were scored with 1–6.5 points^29^, and higher values indicated better postharvest performance^30,31^. Storage duration was quantified as the maximum interval during which 85% of bulbs remained commercially acceptable, indicating a negligible quality decline.

### Statistical analysis

Data were subjected to two-way analysis of variance (ANOVA) to evaluate main effects of organic fertilizer management (Factor A: with and without FMC at 30 t ha⁻¹), nanoparticle treatments (Factor B: sixteen CuO and CaO NPs combinations), and their interactions. The experimental unit was the plot (10.5 m², 150 plants), with block as the replication level (three blocks per season). The statistical model was: *Y*ijk = µ + *FMC*i + *NP*j + (*FMC* × *NP*)ij + *Block*k + ε*ijk*, where *Y*ijk is the observed value, µ is the overall mean, *FMC*i is the organic fertilizer effect, *NP*j is the nanoparticle treatment effect, (*FMC* × *NP*)ij is the interaction effect, *Block*k is the random block effect, and ε*ijk* is the random error term.

The experiment was conducted over two consecutive seasons (2023/24 and 2024/25). The growing season was treated as a fixed effect representing potential year-to-year environmental variation. Preliminary analysis included season as an additional factor in a three-way ANOVA model: *Y*ijkl = µ + *Season*i + *FMC*j + *NP*k + (*Season* × *FMC*)ij + (*Season* × *NP*)ik + (*FMC* × *NP*)jk + (*Season* × *FMC* × *NP*)ijk + *Block(Season)il + εijkl*. Prior to ANOVA, data were tested for normality (Shapiro-Wilk test, *P* = 0.05) and homogeneity of variance (Levene’s test, *P* = 0.05). Percentage data (sprouting, rotting, weight loss) were arcsine square root transformed [arcsin(√*x*/100)] when assumptions were violated, then back-transformed to original units for presentation. Treatment means were compared using Fisher’s least significant difference (LSD) test at *P* ≤ 0.05. Means sharing the same letter designation differ significantly at *P* ≤ 0.05. Pearson correlation coefficients (*r*) were calculated among all measured parameters using treatment mean values. All statistical analyses were performed using R software (version 4.3.0, R Core Team, 2023) with the agricolae package for experimental design analysis.

### Safety and environmental considerations

All procedures for handling and applying nanoparticles adhered to institutional safety protocols aligned with Biosafety Level 2 (BSL-2) standards. This included containment measures, use of appropriate personal protective equipment, and proper waste disposal. Operators wore nitrile gloves, N95 respiratory masks, safety goggles, and long-sleeved protective clothing during nanoparticle preparation and foliar applications. All nanoparticle waste was disposed of in accordance with institutional hazardous materials guidelines. The total Cu applied over four spray events ranged from 40 to 120 g ha⁻¹, significantly lower than traditional Cu fungicides (2–5 kg Cu ha⁻¹ per season) and well below EU limits for Cu in organic farming (6 kg Cu ha⁻¹ annually). Post-harvest soil sampling at 0–30 cm depth revealed that available Cu increased by only 0.15–0.38 mg kg⁻¹ above the baseline of 1.24 mg kg⁻¹, remaining well below the phytotoxicity threshold of over 20 mg kg⁻¹ DTPA-extractable Cu. Final bulb Cu levels ranged from 6.17 to 39.33 mg kg⁻¹ dry weight, all below the Codex Alimentarius maximum for vegetables (40 mg kg⁻¹ DW), ensuring food safety compliance. The total Cu added from FMC at 30 t ha⁻¹ contributed an additional 0.855 kg Cu ha⁻¹, which, combined with nanoparticle applications, remained within safe agronomic limits with minimal risk of environmental accumulation under the tested regime.

## Results

### Analysis of variance for growth, nutrient uptake, and yield parameters

The ANOVA revealed significant main effects and interactions for growth, nutrient, and yield parameters (Table 4). The growing season significantly affected plant biomass (fresh and dry weight) and tissue Cu and Ca concentrations. Organic fertilizer (Factor A) had highly significant effects on fresh weight, dry weight, nitrogen concentration, nitrogen uptake, and tissue Cu concentration. Foliar CuO-CaO NPs treatments (Factor B) significantly affected all measured parameters. Significant A×B interactions were detected for biomass and nitrogen parameters, indicating that the response to nanoparticle treatments differed between organic-amended and unamended plots. Season also significantly influenced bulb yield and mineral concentrations at harvest. Full mean squares and significance levels are reported in Table 4.

**Table 4:** ANOVA for agronomic and physiological traits, yield components, and mineral concentrations under CuO-CaO NPs treatments.

Source of Variation	DF	Fresh Weight	Dry Weight	*N* Concentration	*N* Uptake (g/plant)	*N* Uptake (kg/ha)	Cu (70 days)	
Block	2	8.52^ns^	1.71^ns^	0.04^ns^	0.004^ns^	169.57^ns^	3.27^ns^	
Season (S)	1	61.62***	9.23**	0.09**	0.0004ns	17.65^ns^	17.82**	
Error (a)	2	8.01	0.90	0.01	0.001	45.40	1.59	
Organic Fertilizer (A)	1	18263.12***	12333.32***	75.10***	8.02***	320606.44***	947.41***	
S × A	1	181.92***	23.39***	0.07*	0.0001^ns^	4.02^ns^	70.69***	
Error (b)	4	8.00	0.90	0.01	0.001	45.40	1.59	
Cu-Ca Treatment (B)	15	144.47***	48.66***	0.05***	0.014***	561.24***	1273.84***	
S × B	15	43.11***	6.17***	0.001^ns^	0.002^ns^	69.32^ns^	0.88^ns^	
A × B	15	23.27**	13.04***	0.04***	0.011***	442.09***	22.80***	
S × A × B	15	22.64**	7.05***	0.0004^ns^	0.002^ns^	71.16^ns^	2.49^ns^	
Error (c)	124	8.00	0.90	0.01	0.001	45.40	1.59	

Source of Variation	DF	Ca (70 days)	Chlorophyll (70 days)	Plant Height (70 days)	Bulb Diameter	Bulb Yield	Cu (Bulb)	Ca (Bulb)
Block	2	140698.38***	0.18^ns^	13.69^ns^	0.10^ns^	1.27^ns^	3.11^ns^	421932.93**
Season (S)	1	2156040.19***	22.83***	1.78ns	0.00ns	116.31***	165.95***	1894882.70***
Error (a)	2	10696.49	0.24	12.40	0.20	1.16	2.35	142513.50
Organic Fertilizer (A)	1	103230.75**	22.14***	2.59^ns^	0.01^ns^	233.77***	361.63***	1873485.20***
S × A	1	3245320.02***	0.00^ns^	1.35^ns^	0.00^ns^	0.15^ns^	9.86*	1115995.00**
Error (b)	4	10696.49	0.24	12.40	0.20	1.16	2.35	142513.50
Cu-Ca Treatment (B)	15	4377001.79***	57.31***	19.79^ns^	0.28^ns^	69.34***	501.23***	2982081.50***
S × B	15	136158.98***	1.42***	4.61ns	0.00ns	2.97**	5.73**	218619.40ns
A × B	15	1005341.67***	13.00***	23.93*	0.31^ns^	3.23**	39.20***	1086397.30***
S × A × B	15	174855.17***	1.92***	3.33^ns^	0.00^ns^	2.32*	1.65^ns^	117152.50ns
Error (c)	124	10696.49	0.24	12.40	0.20	1.16	2.35	142513.50

Values represent mean squares from ANOVA. Significance levels: *P ≤ 0.05, **P ≤ 0.01, ***P ≤ 0.001; ns = not significant. DF = degrees of freedom. Error (a) = whole-plot error for season effects; Error (b) = subplot error for organic fertilizer effects; Error (c) = sub-subplot error for Cu-Ca treatment effects. Cu (70 days) and Ca (70 days) represent mineral concentrations in whole plant tissue at the vegetative stage; Cu (Bulb) and Ca (Bulb) represent mineral concentrations in harvested bulbs at maturity.

### Seasonal and organic fertilization effects on growth parameters

The growing season had a significant effect on plant growth parameters (Table 5). Fresh weight was greater in Season 1 than in Season 2 (47.71 g vs. 46.58 g, respectively), as was dry weight. Tissue Cu concentrations were higher in Season 1 (20.53 vs. 19.92 mg kg⁻¹), and Ca concentrations were also higher (3,602.34 vs. 3,390.41 mg kg⁻¹). The FMC application had a pronounced effect on plant nutritional status across all parameters (Table 5). Plants receiving FMC had fresh weights approximately 52% greater than unamended plants (56.90 g vs. 37.39 g) and dry weights approximately 131% greater (28.24 g vs. 12.21 g). Nitrogen concentration was nearly five-fold higher in FMC-treated plants (1.591% vs. 0.340%), which in turn explains the substantially greater nitrogen uptake observed (0.450 g plant⁻¹ vs. 0.042 g plant⁻¹), likely reflecting improved N mineralization in organic-amended sandy soil. FMC-treated plants also accumulated more Cu (22.45 vs. 18.01 mg kg⁻¹), while Ca concentrations were not significantly lower (3,473.19 vs. 3,519.56 mg kg⁻¹), a pattern that may reflect biomass dilution associated with greater growth in organic-amended plots (Table 5).

### Effects of CuO-CaO NPs treatment on growth and physiological characteristics

The fresh-weight responses to CuO-CaO NPs treatments showed clear patterns. The CaO NPs-only applications (Ca1: 51.90 g; Ca2: 51.15 g; Ca3: 51.87 g) were statistically similar to the highest combined treatment (Cu3Ca3: 51.04 g), and all of them were significantly higher than the mineral fertilizer control (40.04 g) (Table 6). The highest dry weights were observed for Ca3 (22.59 g), Ca2 (22.48 g), and Cu3Ca2 (22.05 g), which were not significantly different from each other but showed 44–48% gains over the control (15.72 g). The way nitrogen was used in each treatment differed and was important. The Cu3Ca1 combination had the highest nitrogen content (1.072%), which was not substantially different from Ca1 (1.026%) and Cu1 (1.016%). Treatments with only CuO NPs exhibited significantly lower nitrogen buildup, especially Cu2 (0.833%). The patterns of nitrogen uptake were quite similar to those of concentration. Cu3Ca1 (0.292 g plant⁻¹), Ca1 (0.287 g plant⁻¹), and Ca2 (0.286 g plant⁻¹) were not statistically different from each other, although they were all better than the mineral fertilizer control (0.183 g plant⁻¹).

The amount of Cu in the tissue changed significantly and could be predicted from the application rate. Treatments with 30 mg L⁻¹ CuO NPs had the highest and most distinct concentrations (Cu3Ca3: 36.83 mg kg⁻¹; Cu3Ca2: 35.08 mg kg⁻¹; Cu3: 34.00 mg kg⁻¹; Cu3Ca1: 31.75 mg kg⁻¹). Intermediate treatments ranged from 22.67 to 25.50 mg kg⁻¹. Treatments with CaO NPs only maintained very low tissue Cu concentrations (7.33–10.08 mg kg⁻¹), statistically similar to controls (6.17 mg kg⁻¹). The highest bulb Ca concentrations were observed in high-CaO NPs treatments, particularly when combined with CuO NPs: Cu3Ca3 (4,439.42 mg kg⁻¹), Cu2Ca3 (4,196.33 mg kg⁻¹), and Ca3 alone (4,113.33 mg kg⁻¹), with no significant differences among these three. Moderate combined applications (Cu1Ca2: 3,862.50 mg kg⁻¹; Cu2Ca2: 3,904.58 mg kg⁻¹) also significantly increased tissue Ca relative to lower-input treatments. CuO NPs-only treatments produced the lowest and significantly distinct Ca values (all ≤ 2,730 mg kg⁻¹), indicating potential competitive inhibition or modified transport dynamics (Table 6).

**Table 5:** Mean effects of season, organic fertilization, and CuO-CaO NPs treatments on growth and physiological traits of onion.

Factor	FW (g)	DW (g)	*N* (%)	*N* uptake (g/plant)	Cu/70 day (ppm)	Ca/70 day (ppm)
Season (S)						
First	47.71 a	20.44 a	0.944 b	0.244 a	20.53 a	3602.34 a
Second	46.58 b	20.01 b	0.987 a	0.247 a	19.92 b	3390.41 b
**Organic Fertilization (A)**						
With Organic Fert (FMC)	56.90 a	28.24 a	1.591 a	0.450 a	22.45 a	3473.19 b
Without Organic Fert	37.39 b	12.21 b	0.340 b	0.042 b	18.01 b	3519.56 a
**CuO-CaO NPs Treatment (B)**						
Mineral Fertilizer	40.04 g	15.72 i	0.932 abcd	0.183 e	6.17 i	2982.92 g
CU1 (10 mg L⁻¹)	42.18 fg	16.60 hi	1.016 ab	0.210 cde	14.71 f	2642.50 h
Cu2(20ppm)	46.06 cdef	19.71 fg	0.833 d	0.204 de	22.83 e	2662.50 h
Cu3(30ppm)	46.60 cde	20.69 defg	0.889 bcd	0.226 bcde	34.00 b	2728.75 h
Cu1Ca1 (10 + 50 mg L⁻¹)	44.57 def	17.70 h	0.839 cd	0.193 e	14.83 f	3155.42 f
Cu1Ca2(10 + 100ppm)	43.74 efg	19.36 g	1.003 ab	0.246 abcd	15.92 f	3862.50 c
Cu1Ca3(10 + 150ppm)	46.50 cde	20.49 efg	1.009 ab	0.260 ab	14.33 f	4069.58 b
Cu2Ca1(20 + 50ppm)	49.63 abc	20.41 efg	0.987 abc	0.253 abc	23.33 e	3086.25 fg
Cu2Ca2(20 + 100ppm)	46.62 cde	20.73 cdef	0.966 abcd	0.256 abc	25.50 d	3904.58 c
Cu2C3(20 + 150ppm)	46.29 cde	20.53 efg	0.978 abcd	0.249 abcd	22.67 e	4196.33 b
Cu3Ca1(30 + 50ppm)	47.64 bcde	21.48 abcde	1.072 a	0.292 a	31.75 c	3373.08 e
Cu3Ca2(30 + 100ppm)	48.44 abcd	22.05 abc	0.924 abcd	0.249 abcd	35.08 ab	4071.67 b
Cu3Ca3(30 + 150ppm)	51.04 ab	21.14 bcde	0.999 ab	0.258 ab	36.83 a	4439.42 a
Ca1 (50 mg L⁻¹)	51.90 a	21.92 abcd	1.026 ab	0.287 a	7.33 hi	3085.25 fg
Ca2(100ppm)	51.15 ab	22.48 ab	0.993 ab	0.286 a	8.25 h	3567.92 d
Ca3(150ppm)	51.87 a	22.59 a	0.985 abc	0.283 a	10.08 g	4113.33 b

Different letters indicate significant differences among treatments (*p* ≤ 0.05) according to Tukey’s HSD test. FW: Fresh weight, DW: Dry weight, N %: Nitrogen concentration, N uptake: Nitrogen uptake per plant, Cu/70 day: Cu concentration at 70 days, Ca/70 day: Ca concentration at 70 days.

### Effects of season, organic fertilization, and CuO-CaO NPs treatments on plant height, chlorophyll content, bulb yield, and mineral composition

Height, Chlorophyll Concentration, Bulb Yield, and Mineral Composition.

Chlorophyll content varied between seasons (40.27 vs. 40.96 SPAD), while plant height and bulb diameter (4.90 cm) were relatively stable across seasons (Table 6). Bulb yield was higher in Season 2 (28.82 vs. 27.26 t ha⁻¹), while bulb Cu (13.76 vs. 11.90 mg kg⁻¹) and Ca (3,761.90 vs. 3,563.21 mg kg⁻¹) concentrations were lower, a pattern consistent with biomass dilution. Organic fertilization significantly increased chlorophyll (40.95 vs. 40.27 SPAD), bulb yield (29.14 vs. 26.93 t ha⁻¹), and mineral accumulation (Cu: 14.20 vs. 11.45 mg kg⁻¹; Ca: 3,761.33 vs. 3,563.77 mg kg⁻¹), with no significant effect on plant height or bulb diameter. Among CuO-CaO NPs treatments, chlorophyll content was highest in Cu3Ca3 (44.04 SPAD), which was significantly distinct from all other treatments, followed by Cu3Ca1 (43.65 SPAD) and Cu3 (43.29 SPAD), which were not significantly different from each other. Moderate combination treatments (Cu2Ca1–Cu2Ca3: 41.25–41.54 SPAD) formed a statistically equivalent intermediate group, while CaO NPs-only treatments and the control showed significantly lower values (36.96–38.33 SPAD). Plant height and bulb diameter did not differ significantly across nanoparticle treatments (Table 6).

Bulb yield differed substantially among treatments. Cu1Ca3 (31.78 t ha⁻¹) and Cu2Ca3 (31.52 t ha⁻¹) achieved the highest yields and were not significantly different from each other, but were significantly greater than the control (26.19 t ha⁻¹). Ca3 (30.50 t ha⁻¹), Cu1Ca2 (30.33 t ha⁻¹), and Ca2 (29.63 t ha⁻¹) also significantly exceeded the control. High-CuO NPs treatments without adequate Ca—Cu3Ca1 (23.97 t ha⁻¹), Cu3 (24.61 t ha⁻¹), and Cu3Ca2 (25.30 t ha⁻¹)—yielded significantly less than the best-performing treatments, indicating that 30 mg L⁻¹ CuO NPs may be phytotoxic in the absence of sufficient Ca. Bulb Cu concentration increased with application rate: Cu3Ca3 (24.58 mg kg⁻¹) and Cu3Ca2 (22.83 mg kg⁻¹) were not significantly different from each other and represented the highest group. CaO NPs-only treatments were statistically similar to the control (4.63–7.42 mg kg⁻¹). Treatments with foliar CaO NPs at 100–150 mg L⁻¹ achieved the highest bulb Ca concentrations. The top group (not significantly different from one another) comprised Cu3Ca3 (4,498.50 mg kg⁻¹), Cu2Ca3 (4,347.92 mg kg⁻¹), Cu2Ca2 (4,186.50 mg kg⁻¹), Ca3 (4,109.42 mg kg⁻¹), and Cu1Ca3 (4,162.92 mg kg⁻¹). CuO NPs-only treatments had the lowest bulb Ca concentrations (Cu2: 3,073.08 mg kg⁻¹; Cu1: 3,142.67 mg kg⁻¹; Cu3: 3,151.67 mg kg⁻¹), significantly lower than all Ca-supplemented treatments (Table 6).

**Table 6:** Mean effects of Season, Organic Fertilization, and CuO-CaO NPs Treatments on yield and quality traits of onion.

Factor	Chlo (SPAD)	PH (cm)	BD (cm)	BY (t ha^− 1^)	Cu (ppm)	Ca (ppm)
Season (S)						
First	40.27 b	48.29 a	4.90 a	27.26 b	13.76 a	3761.90 a
Second	40.96 a	48.49 a	4.90 a	28.82 a	11.90 b	3563.21 b
**Organic Fertilization (A)**						
With Organic Fert (FMC)	40.95 a	48.27 a	4.89 a	29.14 a	14.20 a	3761.33 a
Without Organic Fert	40.27 b	48.51 a	4.91 a	26.93 b	11.45 b	3563.77 b
**CuO-CaO NPs Treatment (B)**						
Mineral Fertilizer	36.96 h	46.87 ab	4.67 a	26.19 ef	4.63 i	3066.67 g
CU1 (10 mg L⁻¹)	37.54 h	51.17 a	5.10 a	27.37 de	9.67 f	3142.67 fg
Cu2(20ppm)	41.13 d	48.24 ab	5.17 a	27.09 de	13.75 de	3073.08 g
CU3(30ppm)	43.29 bc	47.78 ab	5.10 a	24.61 gh	20.33 b	3151.67 fg
Cu1Ca1 (10 + 50 mg L⁻¹)	38.58 fg	48.48 ab	4.94 a	28.43 cd	8.50 fg	3381.58 efg
Cu1Ca2(10 + 100ppm)	39.79 e	48.61 ab	4.84 a	30.33 ab	8.83 fg	3942.92 bcd
Cu1Ca3(10 + 150ppm)	41.21 d	46.85 ab	4.96 a	31.78 a	8.92 fg	4162.92 abc
Cu2Ca1(20 + 50ppm)	41.25 d	48.22 ab	4.87 a	28.11 cd	12.42 e	3201.50 fg
Cu2Ca2(20 + 100ppm)	41.42 d	49.45 ab	4.84 a	29.47 bc	17.00 c	4186.50 abc
Cu2C3(20 + 150ppm)	41.54 d	48.85 ab	5.03 a	31.52 a	15.00 cd	4347.92 ab
Cu3Ca1(30 + 50ppm)	43.65 ab	49.25 ab	4.81 a	23.97 h	19.92 b	3423.58 defg
Cu3Ca2(30 + 100ppm)	42.67 c	46.02 b	4.62 a	25.30 fgh	22.83 a	3908.33 bcde
Cu3Ca3(30 + 150ppm)	44.04 a	47.58 ab	4.88 a	26.08 efg	24.58 a	4498.50 a
Ca1 (50 mg L⁻¹)	38.33 g	47.77 ab	4.73 a	28.24 cd	5.17 i	3326.67 fg
Ca2(100ppm)	39.17 ef	49.13 ab	4.97 a	29.63 bc	6.25 hi	3676.92 cdef
Ca3(150ppm)	39.25 ef	49.99 ab	4.87 a	30.50 ab	7.42 gh	4109.42 abc

Different letters indicate significant differences among treatments (*p* ≤ 0.05) according to Tukey’s HSD test. Chlo: Chlorophyll content (SPAD), PH: Plant height, PD: bulb diameter, BY: Bulb yield, Cu: Cu concentration in bulb, Ca: Ca concentration in bulb.

### Seasonal interactions with organic fertilization and CuO-CaO NPs treatments

Both the interaction between growing season and organic fertilizer (S×A) and the main effects of organic fertilizer (A) were significant predictors of plant biomass, whose fresh and dry weights differed across seasons depending on FMC, reflecting how the growth-promoting capacity of organic matter was modified by prevailing seasonal conditions. Cu concentration at 70 days was also significantly affected (*P* < 0.001), indicating that seasonal temperature and radiation patterns exerted different influences on the kinetics of Cu uptake in amended versus unamended soils. Nitrogen concentration showed only a slight S × A interaction (*P* < 0.05), whereas nitrogen uptake parameters were not influenced by the aforementioned treatments. The S×A interaction significantly influenced bulb Ca content (*P* < 0.01) and Cu concentration (*P* < 0.05) at harvest, but accounted for no significant variation in yield or morphological traits, indicating that mineral partitioning to bulbs rather than yield formation was the primary process sensitive to season–organic fertilizer interactions instead of N inputs per se during these years.

The S×B interaction (*P* < 0.001) significantly affected fresh weight, dry weight and Ca concentration at 70 days while the same was true for chlorophyll content (S×B; *P* < 0.001), indicating that the stimulatory effects of foliar Cu-Ca NPs on vegetative growth and chlorophyll content varied with seasonal conditions. This pattern suggests that the efficacy of nanoparticle treatments is sensitive to the prevailing growing environment, reinforcing the value of multi-season evaluation.

The S×A×B interaction had no significant effect on nitrogen parameters, plant height, bulb diameter, or bulb mineral concentrations, indicating that these traits are governed more by individual factor effects than by interactive influences within their seasonal context.

### Multivariate treatment patterns

**Fig. 2:**
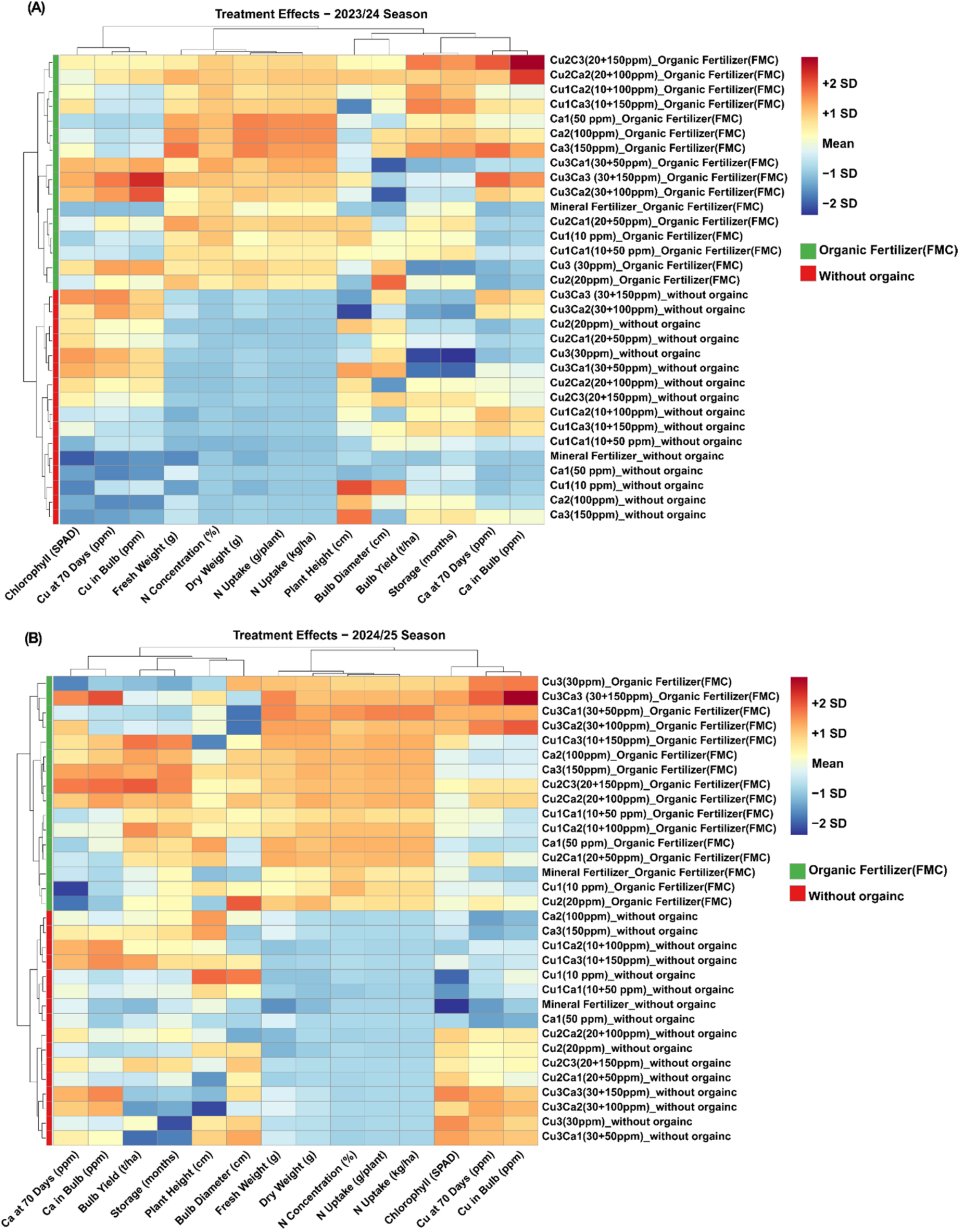
**Hierarchical clustering heatmaps showing treatment effects on growth**,** nutrient uptake**,** and yield parameters in onion cv. Sabeeni across two growing seasons.** (**A**) Season 1 (2023/24) and (**B**) Season 2 (2024/25). Color intensity shows standardized values (z-scores): red for above mean (positive effects), blue below mean (negative), and pale yellow/white near average. Hierarchical clustering on the left (treatments) and top (parameters) shows similarities via Euclidean distance and Ward’s method.

**Fig. 3:**
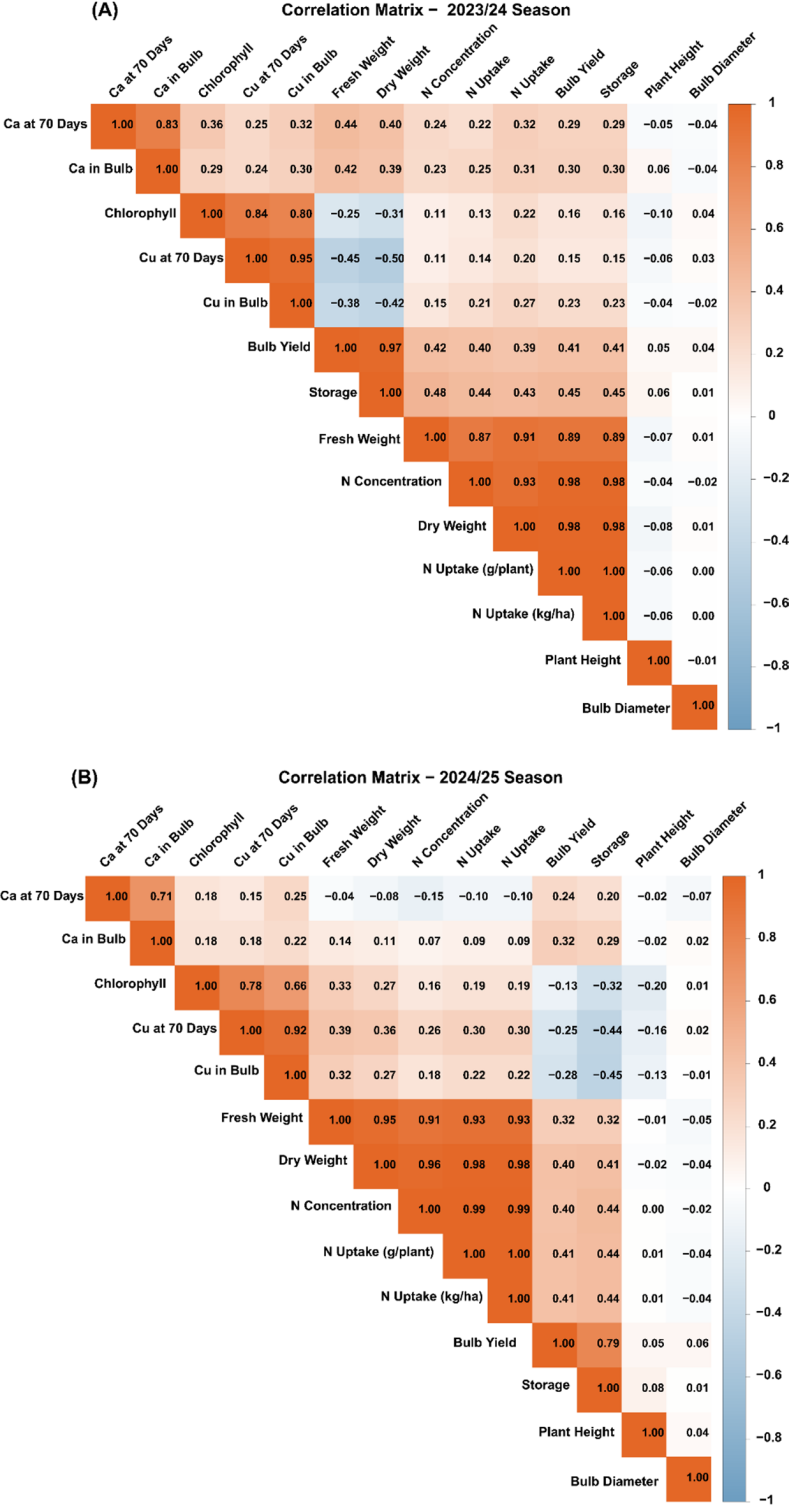
**Pearson correlation matrices showing relationships among growth**,** nutrient uptake**,** and yield parameters in onion cv. Sabeeni across two growing seasons.** (**A**) Season 1 (2023/24) and (**B**) Season 2 (2024/25). Color intensity and numerical values represent correlation coefficients ranging from − 1 (perfect negative correlation, blue) to + 1 (perfect positive correlation, red), with white representing no correlation (*r* = 0). Strong positive correlations (*r* > 0.7) appear in dark orange/red, moderate correlations (*r* = 0.4–0.7) in light orange, weak correlations (*r* = 0.2–0.4) in pale orange/blue, and negligible correlations (|r| < 0.2) in white/pale blue.

Hierarchical clustering identified distinct treatment performance groups (Fig. 2A-B). Treatments combining FMC with moderate-to-high CuO NPs (20–30 mg L⁻¹) and CaO NPs (100–150 mg L⁻¹) clustered together and showed coordinated improvements across vegetative growth, yield, and bulb production parameters, with the top-performing treatments (Cu2Ca3, Cu2Ca2, Cu1Ca2) under organic fertilization showing notably consistent responses. Treatments without organic fertilizer formed a separate cluster with generally lower values across parameters, suggesting that soil organic matter may modulate nanoparticle activity. Mineral fertilizer alone showed intermediate performance (Fig. 2A). Cluster groupings were broadly consistent across seasons, though minor shifts in treatment ranking occurred in Season 2 (Fig. 2B). In the parameter dendrograms, nitrogen, biomass, and yield traits co-clustered, reflecting their functional interdependence (Fig. 3). Ca-related parameters formed a distinct cluster, consistent with Ca’s specific structural and post-harvest roles. The association of storage duration with bulb yield in the same cluster indicates that treatments improving yield also tended to improve post-harvest quality.

### Correlational structure of performance traits

Biomass traits were strongly intercorrelated across seasons: fresh weight and dry weight (*r* = 0.87–0.95), nitrogen concentration (*r* = 0.91–0.99), and nitrogen uptake per plant and per hectare (*r* = 0.89–0.93), consistent with nitrogen availability as a key driver of dry matter accumulation (Fig. 3). The relationship between Cu and other parameters showed some seasonal variation. In Season 1, Cu concentration at 70 days was positively associated with chlorophyll content (*r* = 0.84) and bulb Cu (*r* = 0.95), suggesting efficient Cu translocation to above-ground tissues. Ca showed moderate associations with bulb yield (*r* = 0.24–0.40) and storage duration (*r* = 0.29–0.39), indicating that Ca effects may be expressed primarily during bulb maturation and post-harvest rather than during vegetative growth, consistent with Ca’s structural and membrane-stabilizing roles. Bulb yield was positively correlated with storage duration (*r* = 0.48–0.79), suggesting that treatments supporting greater growth vigor may also improve post-harvest quality. Plant height and bulb diameter showed negligible correlations with other parameters (*r* = − 0.10 to 0.06), indicating that these morphological traits are likely determined by genetic rather than nutritional factors under the conditions studied.

### Integrated performance analysis of nanoparticle and organic fertilizer treatments

**Fig. 4:**
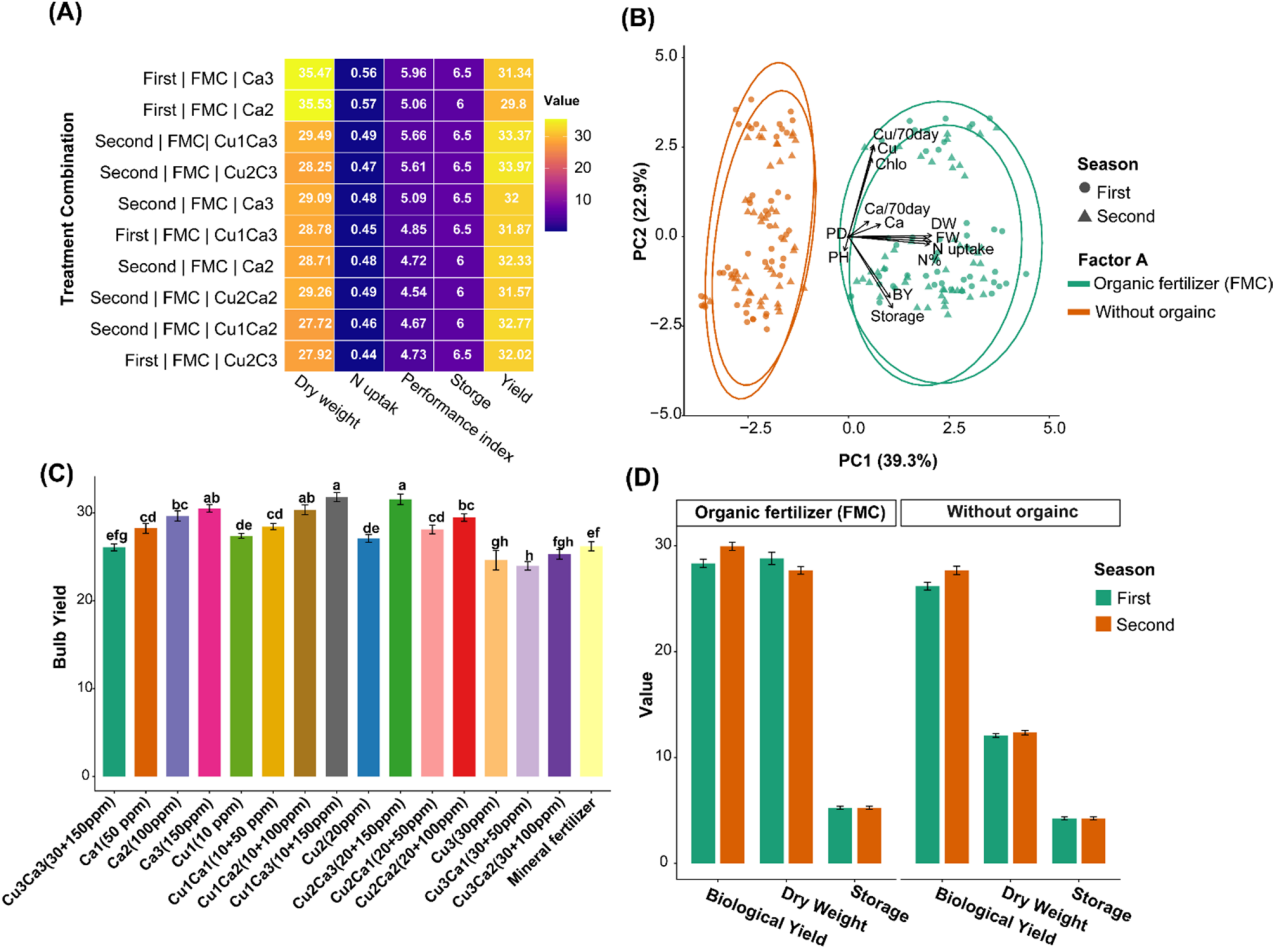
Comparative efficacy of CuO NPs and CaO NPs formulations with organic fertilizer on agronomic performance and quality attributes of onion cv. Sabeeni across two growing seasons. (**A**) Heatmap shows treatment-specific differences in dry weight, nitrogen uptake, performance, storage quality, and yield. (**B**) The principal component biplot displays seasonal variation and treatment clusters, explaining 39.3% (PC1) and 22.6% (PC2) of the variance, with loadings indicating key variables. (**C**) Comparison of bulb yield across CuO and CaO NPs with organic fertilizer; letters mark statistical groups (Tukey HSD, α = 0.05). (**D**) Seasonal stability of bulb yield, dry weight, and storage under organic vs. non-organic fertilizers.

Analysis compares the effectiveness of CuO NPs and CaO NPs at different concentrations with FMC, the heat map in Fig. 4A shows that responses differ with concentration for physiological and agronomic parameters, nitrogen uptake was similar (0.44–0.57), and the performance index ranged from 4.54 to 5.96, showing modulation of photosynthesis and metabolism differed with nanoparticle type and concentration, storage quality was minimally affected (6.6–6.5), suggesting post-harvest longevity is less affected than growth parameters, and yield ranged from 29.8 to 33.4 t ha⁻¹, showing large differences among treatments. PCA (Fig. 4B) reduced the dimensionality of the data, and the first two components captured 61.9% of the variance, with seasonal separation, although some second-season data overlapped with first-season plots, especially for CuO NPs treatments. Vectors show that Ca rate, Cu concentration, bulb yield, and storage were the most important variables, with CuO NPs treatments clustering closely with dry weight and Ca correlating with storage, reflecting their roles in cell wall structure and osmoregulation. Bulb yields (Fig. 4C) ranged from 24.8 to 30.9 t ha⁻¹, with some formulations reaching similar productivity to the fertilizer control. The highest yields were observed with CaO NPs at 100 mg L⁻¹ (Ca1) combined with CuO NPs at 10 mg L⁻¹ (Cu1), while other combinations, such as Cu2 + Ca2 (20 + 150 mg L⁻¹), were not significantly different, indicating that nanoparticle formulations can match conventional approaches under these conditions. Lower doses and certain combinations underperformed, possibly due to antagonism or suboptimal dosing. Seasonal comparison (Fig. 4D) indicated that organic-amended plots maintained relatively stable bulb yields (28–30 t ha⁻¹), whereas non-organic treatments yielded approximately 26 t ha⁻¹ with lower dry weight, suggesting that organic fertility may enhance nanoparticle bioavailability and uptake.

## Discussion

### Organic fertilization as a foundation for nutrient availability

The observed increases in fresh weight (52%) and dry weight (131%) associated with FMC application are consistent with the known benefits of organic matter in arid soils. FMC likely improved nutrient availability through enhanced water retention, cation exchange capacity, and aggregate stability in the sandy substrate^32,33^. The approximately fivefold difference in tissue nitrogen concentration (1.591% vs. 0.340%) likely reflects improved soil N mineralization under FMC, possibly mediated by slow-release organic N, increased moisture retention, or enhanced root development, although root morphology was not measured here. Yield differences between amended and unamended plots are consistent with this interpretation^34,35^. Such results are consistent with previous studies, which have shown that organic amendments tend to be particularly advantageous over mineral fertilizers in sandy, arid soils where rapid nutrient leaching and low cation exchange capacity make the use of minerals less effective^36,37^. Nitrogen uptake reduction of 92–96% caused with and without organic fertilizer (4.33–10.15 vs. climactic level limits by N at an optimal quantity to support all farming practices globally^38^. The interaction between organic amendment and nanoparticle treatment may operate through several mechanisms, including improved foliar absorption due to enhanced plant vigor, organic complexation and stabilization of nanoparticles in the rhizosphere, and a more extensive root system with greater nutrient assimilation capacity^39,40^.

### CuO-CaO NPs interactions and optimal ratios

Cu1Ca3 (10 + 150 mg L⁻¹) and Cu2Ca3 (20 + 150 mg L⁻¹) were identified as the most effective treatments, with yields 15–21% higher than controls, indicating that a combination of moderate CuO NPs and high CaO NPs provides the best nutritional balance, since low-to-moderate CuO NPs (10–20 mg L⁻¹) support photosynthesis, respiration, and antioxidant defenses while high CaO NPs (150 mg L⁻¹) enhance structural integrity by stabilizing cell walls and aiding signal transduction^41,42^. This finding may be particularly relevant to arid regions where native soil Cu levels are adequate, but Ca bioavailability is low due to high soil pH^41,43^.

### Cu concentration thresholds and phytotoxic effects

The high-CuO NPs treatments (30 mg L⁻¹) without adequate Ca balance yielded significantly lower yields (Cu3: 24.61 t ha⁻¹; Cu3Ca1: 23.97 t ha⁻¹) than the optimal low-CuO NPs treatments (Cu1Ca3: 31.78 t ha⁻¹), which were associated with higher tissue Cu accumulation (Cu3: 34.00 mg kg⁻¹; Cu3Ca3: 36.83 mg kg⁻¹). The Cu toxicity thresholds vary widely across plant species, tissues, growth stages, and environmental conditions; in agricultural literature, sensitivity to excess Cu ranges from approximately 15–30 mg kg⁻¹ in sensitive species to 30–100 mg kg⁻¹ in tolerant species^44,45^. The tissue Cu levels observed in the Cu3 treatments (34–36.83 ppm) were above the reported range for moderate sensitivity^46^. The decreased yields at these concentrations, together with increased tissue Cu, suggest phytotoxic effects in onion cv. Sabeeni under the arid conditions of this study, although the mechanism(s) responsible for these effects in the specific experimental system investigated cannot be determined from field observations alone and would require controlled physiological or molecular investigations. The Ca effect on the yield reduction caused by high CuO NPs (Cu3Ca1: 23.97 vs. Cu3Ca3: 26.08 t ha⁻¹) suggests an antagonism between Cu and Ca, which may occur through multiple mechanisms documented in the literature, such as competitive inhibition of plasma membrane nutrient transporters, Ca-mediated reduction of Cu-induced oxidative stress by stabilizing antioxidant enzyme systems, or Ca’s role in maintaining membrane integrity and signal transduction under Cu stress^47,48^. However, the mechanism(s) responsible for the amelioration cannot be determined from field observations alone and would require controlled physiological or molecular investigations.

### Cu’s dual role in photosynthesis and growth

Although Cu functions as a cofactor in photosynthetic electron transport (plastocyanin), the highest chlorophyll values (43.29–44.04 SPAD units) were recorded under high-CuO NPs treatments (Cu3, Cu3Ca1, Cu3Ca3) that had lower bulb yields than moderate-CuO NPs treatments (Cu1Ca1, Cu2Ca1, Cu2Ca3), suggesting a dissociation between early photosynthetic capacity and final economic yield. This pattern may reflect the differential distribution of Cu between leaf and root tissues: foliar-applied Cu may accumulate preferentially in treated leaves and support photosynthetic function, while root-absorbed Cu may exceed tolerance thresholds and impair root growth and nutrient uptake capacity^49,50^ Alternatively, progressive accumulation of Cu over the season may confer early benefits that give way to phytotoxic effects under prolonged oxidative stress. Time-course studies of Cu distribution and tissue-specific physiological responses are needed to evaluate these possibilities. The reversal of the Cu-chlorophyll relationship between Season 1 (positive) and Season 2 (negative) may reflect seasonal modulation of Cu’s physiological effects; higher temperatures and greater evaporative stress during Season 2 could have amplified Cu’s pro-oxidant activity, making phytotoxicity more apparent than under the milder conditions of Season 1. These findings highlight the sensitivity of treatment outcomes to environmental context and underscore the value of multi-season field assessment.

### Ca, post-harvest quality, and storage duration

Storage duration was positively correlated with Ca concentration in harvested bulbs (*r* = 0.39–0.78). The longest storage was observed in Cu1Ca3 and Cu2Ca3 treatments (6.0 and 6.5 months, respectively), compared with approximately 2.0–3.5 months in unamended controls. These findings are consistent with the structural role of Ca in plant tissues^51,52^, which stabilizes the pectin-Ca matrix in cell walls, maintains membrane integrity, delays ethylene production, and inhibits cell wall-degrading enzymes—all of which influence post-harvest longevity. The inverse relationship between tissue Cu concentration and storage duration (*r* = − 0.60) may reflect pro-oxidant effects of excess Cu, promoting lipid peroxidation and accelerating cell senescence, although this interpretation is not definitive since confounding factors (e.g., poorer growth conditions yielding both higher tissue Cu and reduced post-harvest performance) cannot be excluded. The extended storability associated with adequate Ca nutrition (6.0–6.5 months vs. 2.0–3.5 months; *P* < 0.00001) represents a potentially significant economic benefit for smallholder farmers. Extending shelf life by three to four months could reduce post-harvest losses, currently estimated at 30–40% under subtropical conditions^53,54^, thus directly benefiting farm income and market supply.

### Study limitations and future research directions

Although this single-location, two-season design can yield strong within-region estimates of treatment effects, generalizing these results to postharvest reductions in other onion varieties with differing genetics, physiology, or nutrient needs, and arid regions with markedly different soil traits or climate trends may be unwarranted. In addition, the advantage of FMC over mineral fertilization must be analyzed in soil type (sandy loam, pH 8.19, 0.69% organic matter), which has a high organic amendment preference compared to more fertile or finer-textured soils, and that may overestimate the benefit provided by organic amendments if correlated with soils from less leaching potential and higher pH that usually fix mineral nutrients more effectively. Future research should include a variety of varietals and locations to assess generalizability. Controlled physiological and molecular studies are needed to determine the mechanistic basis of several key findings, including: the Cu-Ca antagonism; Ca protection against Cu phytotoxicity; seasonal modulation of Cu effects; and the more specific pathways that link nanoparticle application to an increase in nutrient availability via enhanced root conductance or reduced nutrient excretion; tissue-specific accumulation studies would also be beneficial, along with longer-term sustainability assessments with respect to potential soil Cu accumulation after repeated applications of a given nanoparticle type, heavy-metal cycling throughout the broader agricultural system, and impacts on the soil microbiome arising from whole sustained-powder use or organic matter amendment. Only by integrating a comparative analysis of input costs, increases in yield, halving loses at storage, and longer-term improvements in soil over time can farmers be adequately informed as to whether or not they should adopt such practices.

## Conclusion

This two-season study indicates that integrating organic fertilization with balanced CuO-CaO NPs nutrition is a viable approach for onion production in calcareous hyper-arid soils. The FMC amendment improved vegetative growth and physiological performance (52–131% greater biomass and 92–96% greater nitrogen uptake) and appeared to potentiate subsequent nanoparticle responses. Among the nanoparticle treatments evaluated, foliar Cu1Ca3 (10 + 150 mg L⁻¹) and Cu2Ca3 (20 + 150 mg L⁻¹) produced the greatest marketable yield increases (15–21% over controls) without evidence of phytotoxicity, whereas 30 mg L⁻¹ CuO NPs alone was associated with yield reduction. In soils with adequate native Cu, Ca supplementation at 100–150 mg L⁻¹ alone may be sufficient to support yield gains. Cu and Ca uptake followed dose-dependent patterns, with the highest bulb Ca concentrations (4,109–4,498 mg kg⁻¹ DW) recorded under high-CaO NPs treatments. Correlations between bulb Ca concentration and storage duration (*r* = 0.78–0.87) suggest that pre-harvest Ca nutrition may extend post-harvest storability from 2.0 to 3.5 months to approximately 6.0–6.5 months, with potential implications for reducing the 30–40% post-harvest losses reported in subtropical onion production. Foliar micronutrient application may offer a means of bypassing pH-dependent nutrient fixation in alkaline, calcareous soils, and the combination of organic and nanoparticle inputs may reduce reliance on synthetic micronutrient fertilizers under these agronomic conditions, although long-term soil monitoring and life-cycle assessment are needed before broader environmental conclusions can be drawn. Based on these findings, the following approaches may be considered for arid environments similar to Upper Egypt: FMC at 30 t ha⁻¹ incorporated two weeks before transplanting; foliar nanoparticle applications at 10–20 mg L⁻¹ CuO NPs combined with 150 mg L⁻¹ CaO NPs at 30, 45, 60, and 70 DAT. Validation across additional cultivars and arid environments is needed before large-scale adoption.

## Data Availability

All data generated or analyzed during this study are included in this published article. The datasets used and analyzed in the current study are available from the S.F.L. upon reasonable request.

## Declarations

### Permissions and licenses

Field experiments were conducted at the Agricultural Experimental Farm, Agriculture Research Center, Faculty of Agriculture, Qena University, Egypt, during the 2023/24 and 2024/25 growing seasons. As a faculty member of the Faculty of Agriculture, Qena University, Dr. Abeer Abd EL Moiez Bakr obtained all necessary institutional permissions to conduct this research. The experiments were conducted in accordance with the university’s guidelines and regulations for field research.
